# No Evidence for Emotional Empathy in Chickens Observing Familiar Adult Conspecifics

**DOI:** 10.1371/journal.pone.0031542

**Published:** 2012-02-13

**Authors:** Joanne L. Edgar, Elizabeth S. Paul, Lauren Harris, Sarah Penturn, Christine J. Nicol

**Affiliations:** School of Veterinary Science, University of Bristol, Bristol, United Kingdom; Université Pierre et Marie Curie, France

## Abstract

The capacity of animals to empathise is of high potential relevance to the welfare of group-housed domestic animals. Emotional empathy is a multifaceted and multilayered phenomenon which ranges from relatively simple processes such as emotional matching behaviour to more complex processes involving interaction between emotional and cognitive perspective taking systems. Our previous research has demonstrated that hens show clear behavioural and physiological responses to the mild distress of their chicks. To investigate whether this capacity exists outside the mother/offspring bond, we conducted a similar experiment in which domestic hens were exposed to the mild distress of unrelated, but familiar adult conspecifics. Each observer hen was exposed to two replicates of four conditions, in counterbalanced order; control (C); control with noise of air puff (CN); air puff to conspecific hen (APC); air puff to observer hen (APH). During each test, the observer hens' behaviour and physiology were measured throughout a 10 min pre-treatment and a 10 min treatment period. Despite showing signs of distress in response to an aversive stimulus directed at themselves (APH), and using methodology sufficiently sensitive to detect empathy-like responses previously, observer hens showed no behavioural or physiological responses to the mild distress of a familiar adult conspecific. The lack of behavioural and physiological response indicates that hens show no basis for emotional empathy in this context.

## Introduction

Broad usage of the term empathy covers at least two partially distinct sets of processes; “cognitive empathy” and “emotional empathy” (e.g. [1]). Purely cognitive empathy, also referred to as perspective taking, mentalizing, or Theory of Mind, concerns the capacity to comprehend the viewpoint and/or state of knowledge of another individual, even if this differs from one's own. Emotional empathy concerns the emotional reactions of one individual to the observed experiences of another [2], [3]. Emotional empathy is a multifaceted and multilayered phenomenon which ranges from relatively simple processes such as emotional matching behaviour to more complex events which involve interaction between emotional and cognitive perspective taking systems (*e.g.* see [1]–[6]). While originally considered to be an entirely human phenomenon, there is now evidence that the capacity for emotional empathy, and its sub-components, exists in a range of non-human species [7]. Previous attempts to measure empathy in animals have focussed on using behavioural or physiological measures of stress (see [7] for a review). Our previous research employed both behavioural and physiological parameters, demonstrating that mother hens (*Gallus gallus domesticus*) responded to their chicks' distress with increased heart rate, stress-induced hyperthermia and increased vocalisations. This indicates that they are responsive to their chicks' distress and possess the foundations of emotional empathy [8]. It has been hypothesised that empathy evolved to facilitate parental care and it is likely to be adaptive for animals to be affected by the distress of their offspring [5]. The extent to which chickens are affected by unrelated, yet familiar, conspecifics is unknown but is a question of relevance to commercial farming situations, in which the distress of conspecifics may be witnessed routinely, during handling, transport and slaughter.

Using established methodology [8] we aimed to gain information on the behavioural (behaviour and vocalisations) and physiological (heart rate, heart rate variability, eye and comb temperature) responses of observer hens whilst witnessing familiar adult conspecifics being exposed to regular puffs of air, a mildly aversive stimulus for chickens (i.e. known to generate behavioural and physiological signs of distress [8]). As well as general behaviour, heart rate and heart rate variability were used as indicators of sympathetic and parasympathetic nervous system activation. Additionally, surface temperatures of the eye and comb were used to detect possible stress-induced hyperthermia. Existence of such a response could be used to determine the capacity for emotional empathy in chickens outside the mother/offspring bond.

## Methods

### Statement of ethical approval

This project was carried out following ethical approval by the University of Bristol (University Investigation Number: UB/07/002).

### Animals and housing

36 female chickens (Lohman brown, aged 24 weeks) were obtained from a commercial laying hen rearer. The hens were housed in groups of six in floor pens (3 m×2 m), bedded with 5 cm of wood shavings, containing nest boxes and perches. Each of the six pens contained two observer hens and four conspecific hens. Each of the 12 observer hens was assigned two conspecific hens, one for the current study and one for a subsequent study not described here. The temperature in the room was 20°C and the lighting schedule was 12L∶12D. *Ad libitum* layers' mash was provided from a suspended feeder and water from a suspended drinker. Upon arrival, each hen was leg tagged for identification and the hens were allowed to familiarise themselves with each other for three weeks before habituation.

### Week 1: Habituation

Each day, for five consecutive days, each observer hen and her designated conspecific were habituated to the test procedure and apparatus. To prepare hens for non-invasive heart rate monitoring each bird was fitted with a harness. The harness was made from elastane, fitted around the back and tail, between the legs and secured behind the neck with hook and loop fastenings, allowing free limb movement. A pocket for holding the heart rate monitor was positioned over the hen's back. On days 4 and 5 self-adhesive electrode sensors (Ambu Blue sensor M-00-S) were applied before fitting the harness; each hen was gently placed on her back, two small sections of skin overlying the pectoralis muscle either side of the sternum were cleaned using surgical spirit and cotton wool, and the electrode sensors were applied to the cleaned skin. On day 5, a non-invasive telemetric logging system [9] was placed in the pocket and connected to the sensors on the hen's skin using two attached wires.

After the harness fitting process on each habituation day, the observer and conspecific hens were placed in the test apparatus and left undisturbed for a period of 20 minutes. The test apparatus was a 100 cm×50 cm wooden structure divided into two sections; the Observer Box and the Conspecific Box, which were separated by a clear perspex screen. After the 20 minute period, the hens were removed from the apparatus and the harness was removed from the observer hen.

### Weeks 2–3: Testing

Observer hens were tested on consecutive days for four days per week, for two weeks, making up two replicates. Each hen was assigned to one treatment condition (Control, C; Control with noise, CN; Air puff to conspecific hen, APC; Air puff to observer hen, APH) per day of testing, such that hens experienced all four conditions in a randomised order, across each consecutive four-day testing replicate.

Each day, observer hens were fitted with the heart-rate monitor (see above) then placed into the Observer box. The hen's conspecific was then placed into the Conspecific box and a 5 minute settling period commenced. After this, hens were exposed to one of four conditions:

#### 1) Control (C)

Observer hen and conspecific were left undisturbed for a period of 20 minutes. For consistency with the other three conditions during analysis, this condition was split into a notional 10-minute pre-treatment period and a 10-minute treatment period, although no specific treatment was applied.

#### 2) Control with noise of air puff (CN)

After the 10-minute pre-treatment period, a 10-minute treatment period began in which the air puff was sprayed from the same location as APH (see below) but was directed away from both the Observer and Conspecific boxes, so that the hen could hear the noise of the air puff (identical to the sound of a household aerosol being sprayed). This occurred for one second every 30 seconds.

#### 3) Air puff to conspecific hen (APC)

After a 10-minute pre-treatment period, during which the hen and conspecific were left undisturbed, a 10-minute treatment period began in which an air puff from a canister of inert compressed air (Sprayduster, AF International, UK) was sprayed into the Conspecific box for one second every 30 seconds.

#### 4) Air puff to observer hen (APH)

After the 10-minute pre-treatment period, a 10-minute treatment period began in which the air puff was sprayed into the Observer box for one second every 30 seconds.

Immediately after each test, both hens were removed from the apparatus, the harness and heart rate monitor was removed from the observer, and the observer and conspecific hens were returned to their home pen.

### Physiological and Behavioural Responses

Physiological and behavioural parameters were monitored throughout the pre-treatment and treatment periods for all four conditions.

### Physiology

A thermal imaging camera (ThermaCam E4, FLIR) was used to capture a thermal image of the observer hen's head every minute. Maximum eye and comb temperature were obtained using ThermaCAM Reporter 2000 Professional. A time window of five seconds at either side of the one-minute mark was allowed to ensure that a clear image of the side of each hen's head was obtained. Distance from the hen was maintained at one metre and the thermal camera set to an emissivity of 0.96. The ambient temperature of the testing room was maintained at 20°C.

Heart rate data were obtained and analysed using a non-invasive telemetric logging system and software [9]. Heart rate variability was calculated using Spike 2 (Cambridge Electronic Design, UK).

### Behaviour

Observer hen behaviour and vocalisations, and conspecific alarm and warning vocalisations were recorded continuously using a video camera positioned over the test apparatus. Videos were analysed using *Observer* 5.0 (Noldus, Nottingham, UK). All the behaviours and vocalisations listed by previous authors [10], [11] were included, using the same descriptors.

### Statistical analyses

Heart rate (bpm – beats per minute) was calculated every minute and heart rate variability (RMSSD - square root of the mean of the sum of the squares of differences between successive inter-beat intervals, and SDNN – standard deviation of the inter-beat intervals) was calculated every two minutes. Heart rate, heart rate variability and temperature data were averaged over each 10-minute period (pre-treatment and treatment) to produce one data point per 10-minute period. The duration (percentage of time) of all performed behaviours and vocalisations were calculated for each 10-minute period.

Data were analysed using *PASW Statistics 18* (IBM, Portsmouth, UK). All data were checked for normality using a Kolmogorov-Smirnov test and all non-normal data were transformed using the formula *x* = (*x*+0.5)^0.5^. A repeated measures ANOVA was conducted with Condition (C, CN, APC, APH) and Period (pre-treatment, treatment) as within-subjects factors. Post-hoc tests (LSD) were conducted in the event of a significant interaction effect from an ANOVA, during which pre-treatment and treatment periods were compared for each condition. A mixed between-within subjects model was used to consider the influence of an additional factor; the order of testing and hence the prior experience of the hens (*i.e.* whether hens experienced *i*) APC before APH (*n* = 7) or *ii*) APH before APC (*n* = 7)). Spearman's rank order correlations were used to test for associations between hen vocalisations and conspecific vocalisations.

## Results

### Physiological recordings

During treatment conditions C and CN, there were no significant differences between the pre-treatment and treatment periods for heart rate, heart rate variability and comb temperature.

**Figure 1:**
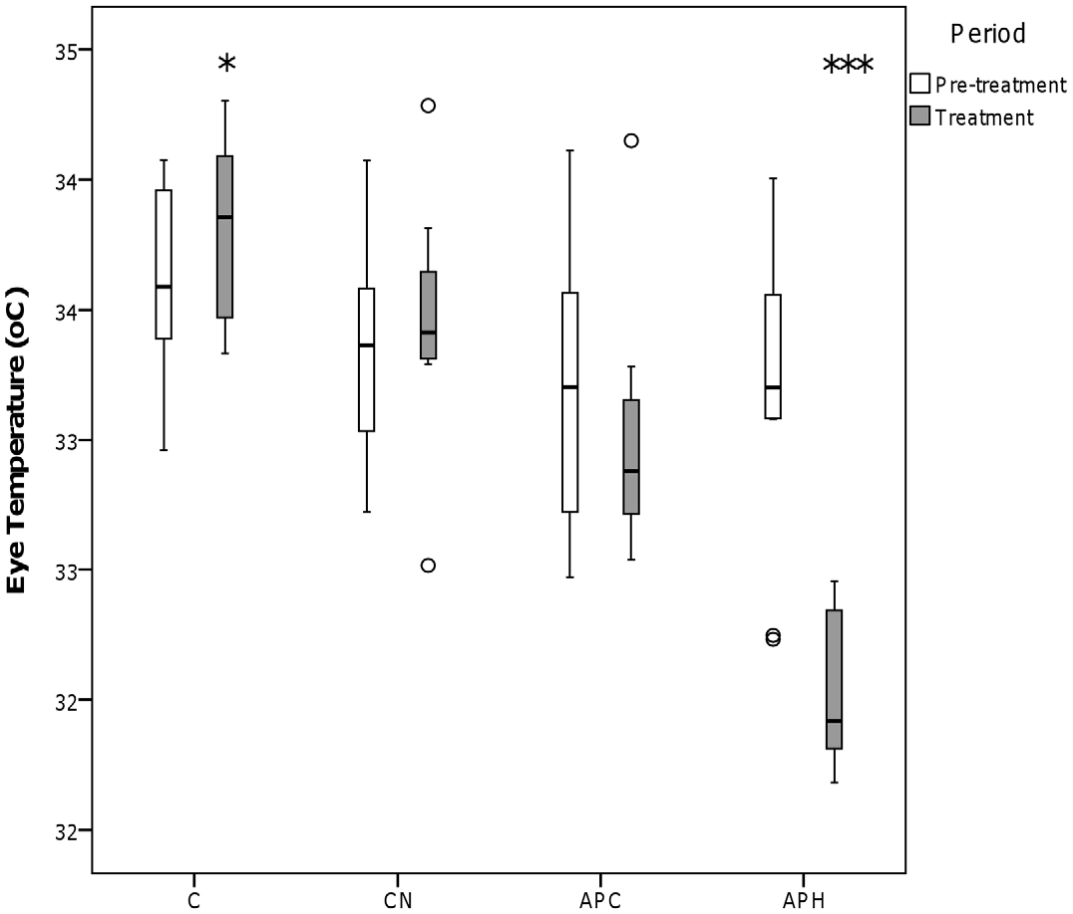
Eye temperature (°C) of the hens during the four conditions. C = Control, CN = Control with noise APC = Air puff to chicks, APH = Air puff to observer hen. ‘°’ represents outliers >1.5 times the interquartile range and ‘*’ represents outliers >3 times the interquartile range. * *p*<0.05, ** *p*<0.01, *** *p*<0.001.

**Figure 2:**
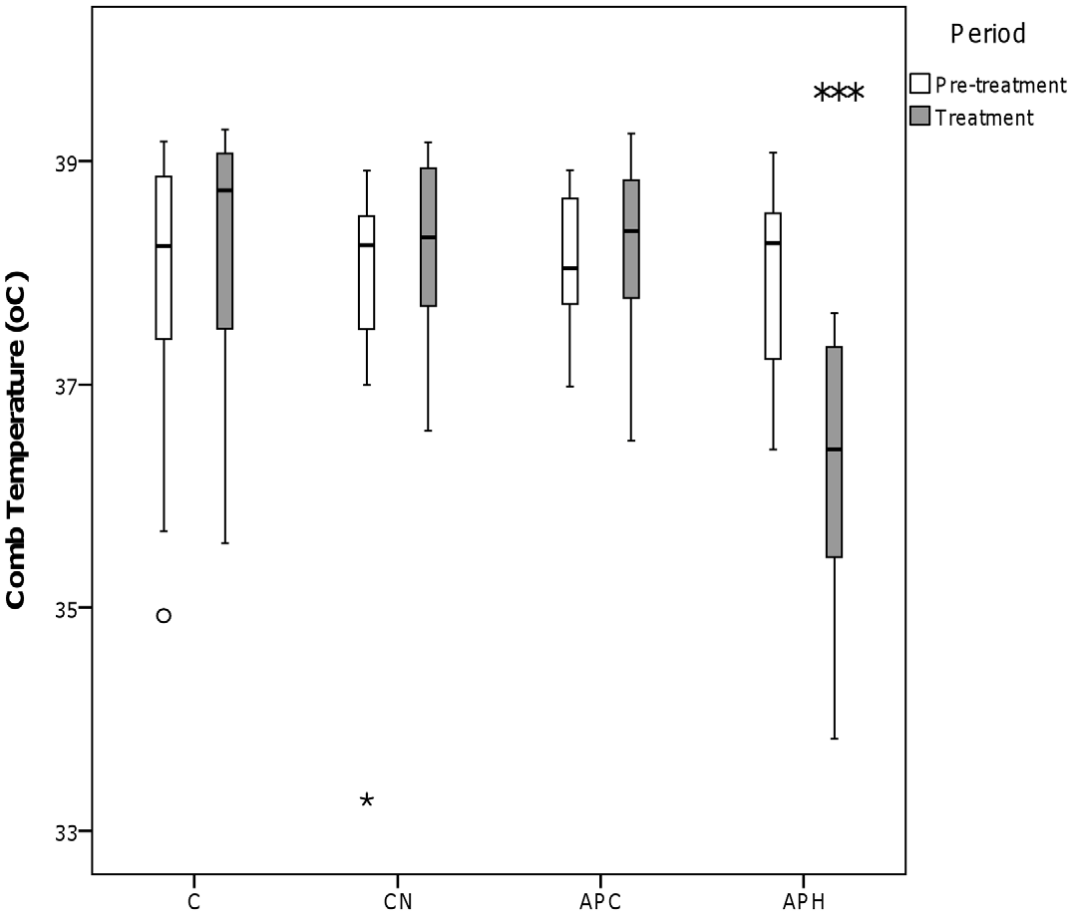
Comb temperature (°C) of the hens during the four conditions. C = Control, CN = Control with noise, APC = Air puff to conspecific, APH = Air puff to observer hen. ‘°’ represents outliers >1.5 times the interquartile range and ‘*’ represents outliers >3 times the interquartile range. * *p*<0.05, ** *p*<0.01, *** *p*<0.001.

A significant interaction effect between Condition and Period was noted for eye temperature (see Fig. 1) (Wilks Lambda = 0.160, *F*_3,9_ = 15.710, *p* = 0.001) and comb temperature (see Fig. 2) (Wilks Lambda = 0.048, *F*_3,9_ = 60.014, *p*<0.001). Specifically, in the APH condition, eye and comb temperature decreased by approximately 2°C between the pre-treatment and treatment period. Conversely, there was a very slight increase in eye temperature between the pre-treatment and treatment periods in the C condition (see Fig. 1).

There were no significant interactions between Condition and Period for heart rate or heart rate variability.

### Behavioural observation*s*

There were no significant differences between the pre-treatment and treatment periods for any behaviour during condition C.

**Figure 3:**
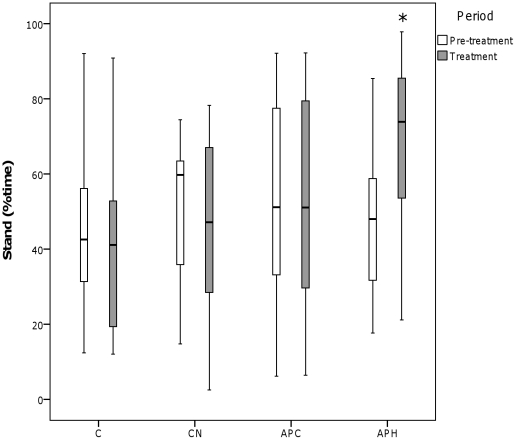
Percentage time spent standing during the four conditions. C = Control, CN = Control with noise, APC = Air puff to conspecific, APH = Air puff to observer hen. ‘°’ represents outliers >1.5 times the interquartile range and ‘*’ represents outliers >3 times the interquartile range. * *p*<0.05, ** *p*<0.01, *** *p*<0.001.

**Figure 4:**
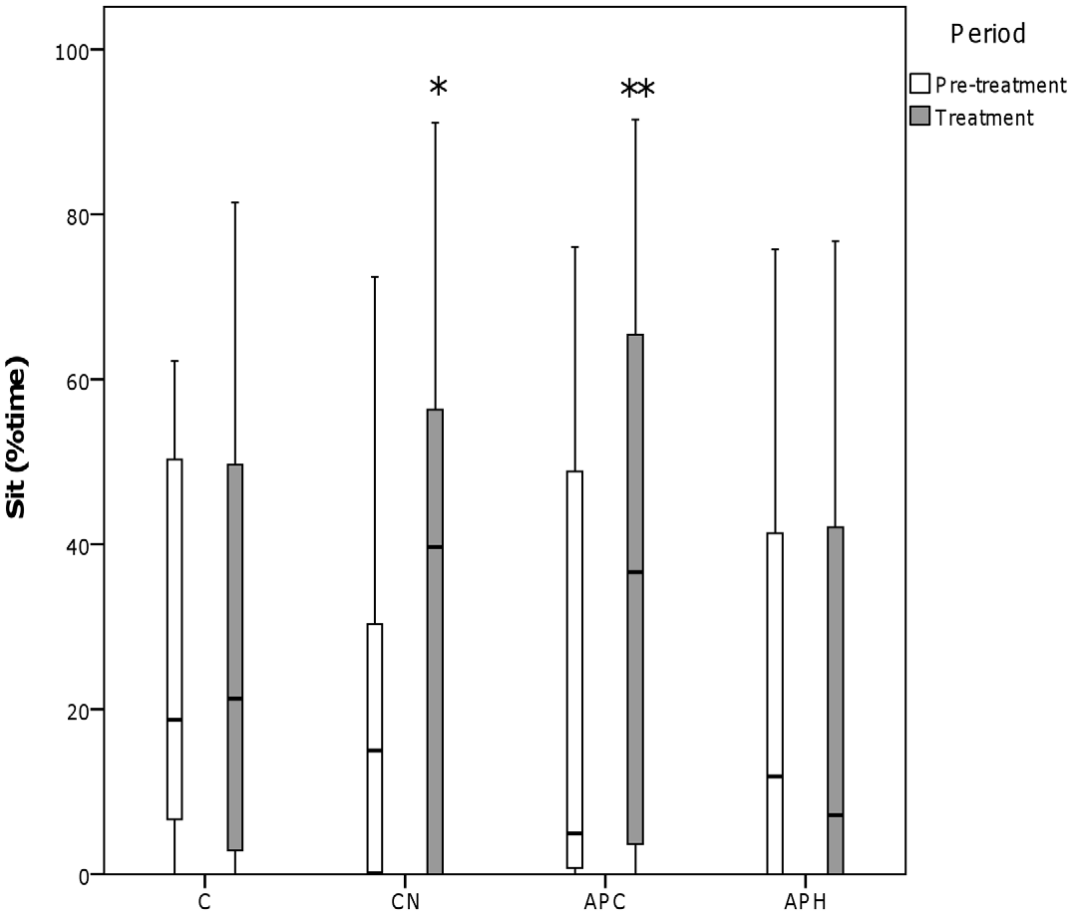
Percentage time spent sitting during the four conditions. C = Control, CN = Control with noise, APC = Air puff to conspecific, APH = Air puff to observer hen. ‘°’ represents outliers >1.5 times the interquartile range and ‘*’ represents outliers >3 times the interquartile range. * *p*<0.05, ** *p*<0.01, *** *p*<0.001.

There was a significant interaction between Condition and Period for standing alert (see Fig. 3) (Wilks Lambda = 0.351, *F*_3,9_ = 5.538, *p* = 0.020) and sitting (see Fig. 4) (Wilks Lambda = 0.358, *F*_3,9_ = 5.369, *p* = 0.021), with an increase in standing alert between the pre-treatment and treatment periods for APH and an increase in sitting between the pre-treatment and treatment periods for CN and APC.

There was no significant interaction between Condition and Period for ground pecking, preening, walking, warning calling, alarm calling or any other behaviour. Similarly, there were no significant interaction effects for total number of behaviours, time spent in different areas of the Observer box or movements between the different areas of the box.

### Order of testing

The order of testing had no influence on the hens' response to the conditions.

### Conspecific warning and alarm vocalisations

For conspecific warning and alarm vocalisations, there was no interaction effect between Condition and Period. During the APC condition, conspecific hens spent 1.2% of their time emitting warning vocalisations, compared with 0% during C, CN and APH conditions. Conspecifics spent 1.3% of their time alarm calling during APC, compared with 0%, 1% and 0% during C, CN and APH respectively.

## Discussion

During the Control condition (C), no significant changes were observed in hen behaviour between the pre-treatment and treatment periods. The only change noted during C was a very slight increase in eye temperature between the pre-treatment and treatment periods. The Control with noise of air puff (CN) condition had similarly little effect, indicating that extraneous stimuli associated with the air puff (*e.g.* noise of puffing action) did not, by themselves, have any manifest effect on the hens, either directly or associatively via a learned connection with the air puff sensation itself.

During the observer hens' exposure to the air puff (APH), eye and comb temperature decreased and time spent standing alert increased. Decreased surface body temperature is a likely indicator of peripheral vasoconstriction, part of a broader process of stress-induced hyperthermia, a phenomenon documented in rats [12], sheep [13], and chickens [8]. In domestic chickens, increased time spent standing alert is associated with fear in response to novel object, open field and predator tests [14] and hens are known to selectively avoid environments associated with high levels of standing alert [10]. The behavioural and physiological responses of the observer hen in the APH condition were very similar, or even greater (in the case of temperature change) to the equivalent treatment in our previous study which considered mother hens' responses to their chicks receiving an air puff [8]. This indicates that the air puff fulfilled its role as a stressor. Taken together, the responses during APH in the current study are likely to indicate generalised physiological arousal and increased vigilance [8]. In the previous study, the hens' attention might have been focussed on their chicks, rather than themselves, even during the APH treatment, thereby decreasing the behavioural and physiological effects of the treatment on the observer birds themselves.

During the conspecifics' exposure to the air puff (APC), the only response detected in the observer hens was an increase in sitting. This did not occur during any of the other treatments and it is unclear why these hens showed increased sitting behaviour. There was no indication of heightened physiological arousal and the sitting posture more likely indicated that hens were in a calmer, more ‘relaxed’ state. This result is in direct contrast to our previous findings when hens responded to air puffs directed at their chicks with increased alertness and maternal vocalisations [8].

The lack of response during the APC treatment for adult conspecifics indicates no basis for emotional empathy in terms of any distinction being made by observer hens between this condition and the control conditions. It has been hypothesised that empathy evolved to facilitate parental care, with it being adaptive for animals to be emotionally affected by the distress of their offspring [5]. It is possible, therefore, that although hens are affected by the distress of their chicks, they show no form of emotional empathy or emotional matching outside the mother-offspring bond. However, responses to a familiar conspecific's pain or distress do seem to extend outside the mother-offspring bond in rodents, with mice showing enhanced pain- [15] and distress- [16] related behaviour after witnessing a familiar conspecific in pain (injected with a pain-inducing substance [15] or subject to repetitive foot shocks [16]) and further research is needed before this capacity can be completely ruled out in chickens.

Despite following essentially the same methodology as our previous study of mother hens and their chicks [8], there were some unavoidable differences between the two studies. In the current study, hens observed a single conspecific in distress, compared to a group (between four and eight) of their own chicks in the previous experiment. This allowed us to use the same sized conspecific box in each experiment. However, it leaves open the possibility that chickens might respond empathically only to a group of several conspecifics rather than to an individual; if a number of conspecifics are showing signs of distress then this may be more likely to be an honest indicator of threat, worthy of generating an emotional response in an observer. Another possibility is that hens might respond empathically to adult conspecifics if these were more closely related e.g. sisters previously brooded together.

It could be argued that the air puff was too mild a stressor for an adult conspecific to generate an empathic response in the observer hen; despite the hens responding to the stressor directed at themselves, the response may have been too small to facilitate social transfer and generate an empathic response. However, conspecifics in the current study actually produced slightly more alarm and warning vocalisations than the chicks in the previous study produced distress vocalisations [8]. This suggests that these adults could have been affected by the air puff to a *greater* degree than chicks were previously. However, chick distress vocalisations might not serve exactly the same function as adult alarm and warning vocalisations so no direct comparison can be made. Whatever the explanation, the result clearly shows that empathic responses in hens are not facilitated by warning or alarm vocalisations. The mother hens' responsiveness to the APC (air puff to chick) condition in our previous study [8] may have been mediated via the hens' perceptions of a threat to their chicks, rather than via their detection of chick distress vocalisations.

It is also important to consider that the parameters measured in the current study were not exhaustive, and there is a possibility that observer hens might have responded in other ways, *e.g.* hormone changes, facial expressions, subtle vocalisations or changes in posture. Future empathy studies should aim to measure as many potential indicators of arousal as is feasible, and in contexts which involve husbandry-relevant stressors.

The absence of distinct behavioural and physiological responses in hens observing mildly distressed conspecifics indicates that, using the measured parameters, there is no evidence of any form of emotional empathy in chickens in the present context. Further research is needed to determine whether chickens show emotional empathy with adult conspecifics in the context of more severe or more salient and husbandry-relevant stressors, or when witnessing the distress of related, as opposed to unrelated, individuals.
